# Evidence for a new species of *Cryptosporidium *infecting tortoises: *Cryptosporidium ducismarci*

**DOI:** 10.1186/1756-3305-3-21

**Published:** 2010-03-25

**Authors:** Donato Traversa

**Affiliations:** 1 Department of Comparative Biomedical Sciences, University of Teramo, Italy

## Abstract

Cryptosporidiosis affects the gastrointestinal and respiratory tract of humans as well as of a wide range of companion, farm, laboratory and wild animals. In the past few years, three independent studies have provided strong evidence for the existence of a distinct *Cryptosporidium *species affecting tortoises and likely circulating in other reptile species as well. A new *Cryptosporidium *genotype was firstly detected and genetically characterized in a marginated tortoise in Italy in 2007 and named *Cryptosporidium *sp. ex *Testudo marginata *CrIT-20. The phylogenetic analysis of this isolate indicated that this *Cryptosporidium *was unique and belonged to the intestinal clade. These findings were later on confirmed by the detection of genetic homologies of isolates from a python and a chameleon from Spain and by recent research in the United States. The latter study presented both the occurrence of intestinal lesions in a pancake tortoise and a Russian tortoise and the genetic characterization of the isolates, together with the first pictures of the endogenous stages of *Cryptosporidium *CrIT-20. Phylogenetic inference based on the sequences representing small subunit of the nuclear ribosomal RNA gene (SSU) of these isolates confirmed the pathological findings because this *Cryptosporidium *was related to the intestinal group and supported previous results in *T. marginata *from Italy. The present scientific data on the *Cryptosporidium *CrIT-20 support its classification as a new species of *Cryptosporidium *causing intestinal diseases in tortoises. Although further morphological (i.e. exogenous stages) and biological aspects (i.e. complete host range) are yet to be elucidated, it is proposed that this *Cryptosporidium *is designated *Cryptosporidium ducismarci*.

## Findings

This report aims to propose a new species of *Cryptosporidium *isolated from reptiles. *Cryptosporidium *spp. are apicomplexan parasites of a wide range of animals. Due to their biology, ecology and epidemiology these protozoa are globally distributed. The vertebrate hosts become infected through host-to-host contact or through ingestion of contaminated food or water [1,2]. The taxonomy of *Cryptosporidium *has been debated and several doubts and uncertainties still exist. For a long time the only recognized species have been *Cryptosporidium parvum *and *Cryptosporidium muris*. However, numerous other isolates were present in animals but described only in the last decades [3]. The difficulties in addressing *Cryptosporidium *taxonomy and in delineating new species mainly rely on the inability to morphologically discriminate the biological stages and on the difficulties in establishing monospecific experimental infections [3]. With the advent of nucleic acid-based techniques and sequencing, important results have been achieved in elucidating the taxonomical status of several *Cryptosporidium *isolates and presently 19 distinct species are known to affect amphibians, reptiles, birds and mammals [2,4]. Three more species are hypothesized to infect fishes but their status is not supported by sound biological and molecular data [2,4]. Indeed, the genetic characterization, at one or more loci, of *Cryptosporidium *is a fundamental requirement to assess, at the species level, a new *Cryptosporidium *isolate, along with the morphological description of the biological stages and definition of the host range, possibly based upon natural and experimental infections [2,4].

Non-conventional animals, such as reptiles, have increased in popularity as pets in developed countries, posing new concerns in relation to their pathogens. Despite reptile cryptosporidiosis being recognized since the 1970s [5] the first described species (i.e. *Cryptosporidium serpentis*), which is able to cause significant gastric pathologies in snakes, has been genetically described only recently [6,7]. Subsequently, *Cryptosporidium varanii/saurophilum*, whose taxonomic identity has been debated for some time, has been elevated as a proper species of intestinal pathogen of lizards [2,8]. Beyond these two species, other isolates have been reported sporadically and partially described from different reptile species [9-11]. Indeed, our understanding of *Cryptosporidium *affecting chelonians is very limited. The reports of *Cryptosporidium *spp. in Testudines published in the last fifteen years relate to the infection in the following species: radiated tortoise (*Geochelone radiata*), Indian star tortoise (*Geochelone elegans*), travancore-like tortoise (*Indotestudo *spp.), gopher tortoise (*Gopherus polyphemus*), spur-thighed tortoise (*Testudo graeca*), Egyptian tortoise (*Testudo kleinmanni*) and Hermann's tortoise (*Testudo hermanni*) [9,10,12,13], but no comprehensive data on multi-locus genetic characterization, infection site/s or biological aspects were generated. For example, in a study performed in 1997 in the United States, *G. radiata*, *G. elegans*, *Indotestudo *sp. and *G. polyphemus *were found positive at microscopic and immunofluorescence assays [13] which, however, have important limits in terms of specificity and do not allow a discrimination at the species/genotype level. Another key example is provided by the fatal intestinal pathology ascribed to *Cryptosporidium *spp. in *T. kleinmanni *in 1998, which was not supported by the genetic characterization of the pathogen [12].

In the past few years, three independent studies have provided strong evidence for the existence of a distinct *Cryptosporidium *species affecting tortoises and probably circulating in other reptile species as well. The first clue for the presence of this species was published in 2008: a genetic isolate of *Cryptosporidium *has been found and characterized from the faeces of a marginated tortoise (*Testudo marginata*) living in an open enclosure of central Italy [14]. A multi-locus genetic analysis *via *the characterization of informative regions within the genes encoding the *Cryptosporidium *oocyst wall protein (COWP) and the small subunit of the nuclear ribosomal RNA (SSU rRNA) was conducted. The COWP and SSU sequences revealed only ~80% and ~94% identity with the corresponding sequences of the ubiquitous *C. parvum *(GenBank™ Accession Numbers XM_627569 and AF108864 AF108864). Molecular and phylogenetic analyses clearly demonstrated that this isolate was an unique uncharacterized genotype, which has been named *Cryptosporidium *sp. ex *Testudo marginata *CrIT-20. The phylogenetic inference showed this isolate had a new COWP sequence at the interface of the two major sister groups (i.e. intestinal *C*. *parvum *and gastric *C. muris*). Further analysis of the COWP amino acid sequence for *Cryptosporidium *CrIT-20 showed partial common residues with the gastric *C. muris *(79% nucleotide identity), as well as, with avian *Cryptosporidium baileyi *(81% nucleotide identity) affecting the intestinal and respiratory tract of birds. Also, phylogenetic analysis of the SSU rejected the possibility that *Cryptosporidium *CrIT-20 is the star tortoise genotype [10,14] and confirmed that this genotype is monophyletic with the intestinal *Cryptosporidium *species [14]. More specifically, the phylogenetic trees showed CrIT-20 genotype being at the bottom of the intestinal species, together with a diverse spectrum of reptilian and avian genotypes and of *C. baileyi *[14]. The hypervariable region of the SSU sequence of *Cryptosporidium *CrIT-20 (corresponding to the nucleotides 633-703 of *C. parvum *GenBank™ Accession Number AF108864) revealed high genetic differences when compared with the same sequence of other 28 *Cryptosporidium *species/genotypes, supporting the CrIT-20 unique position [14].

A second distinct survey performed in 2008 showed the presence of *Cryptosporidium *CrIT-20 in the faeces of a chameleon (*Chamaeleo calyptratus*) and a python (*Python regius*) in Spain [11]. The neighbour-joining analysis of partial SSU sequence data confirmed this *Cryptosporidium *clustering with the species affecting the intestine but representing a distinct clade. Nonetheless, the characterization of a fragment within the gene encoding the 70 kDa heat shock protein indicated this *Cryptosporidium *to be related to the gastric species, highlighting the need of further more-in-depth study to identify the site of infection. Following the first molecular detection of *Cryptosporidium *CrIT-20 and its recognition as an unique genotype in *T. marginata *[14], the Spanish researchers claimed that "a new *Cryptosporidium *species or genotype closely related to the main intestinal *Cryptosporidium *species was isolated from a chameleon and a python. This one was found to be related to a further new *Cryptosporidium *sp. recently identified in a tortoise" [11]. Indeed, the molecular comparison of the SSU sequences generated in Italy [14] and Spain [11] showed a single nucleotidic difference (i.e. a thymine insertion) confirming that the Italian and the Spanish isolates belonged to the same *Cryptosporidium *genotype. In fact, given the nature of the SSU gene, such a mutation is not taxonomically relevant given that different copies of the SSU gene may have minor intraspecific differences in *Cryptosporidium *spp. [3].

A third and last study provided new evidence for the existence of a novel species of *Cryptosporidium *in reptiles [15]. A group of researchers from the USA documented two cases of fatal intestinal cryptosporidiosis in a *Malacochersus tornieri *(pancake tortoise) and in a *Testudo horsfieldii *(Russian tortoise) and presented both the occurrence of gut lesions and the genetic characterization of the isolate. The necropsy of the two animals showed intestinal lesions consistent with cryptosporidiosis (i.e. mucosal alteration of the *lamina propria *and its infiltration with lymphocytes, plasmacells and heterophils). This study [15] provided the first pictures of the intestinal stages of this genetic type of *Cryptosporidium*. Specifically, numerous round *Cryptosporidium *stages, of 1-2 μm of diameter, were found closely adhering to the brush border along the intestinal mucosa of the Russian tortoise and, analogously, various *Cryptosporidium *stages with a diameter ranging from 1 to 5 μm were present within the cytoplasm of intestinal cells and admixed with mucous in the pancake tortoise as well. Given that SSU is the most investigated genetic locus to address taxonomic questions within the Apicomplexa, including *Cryptosporidium*, this locus was characterized also for these isolates [15]. Interestingly, the SSU region of the *Cryptosporidium *isolate from the *M. tornieri *and the *T. horsfieldii *in the USA [15] showed a 100% homology to the sequence of *Cryptosporidium *CrIT-20 from Italy [14]. The phylogenetic inference confirmed the pathological findings (i.e. site of infection and disease), as the US *Cryptosporidium *was strongly related to the intestinal group of *Cryptosporidium *spp. and segregated in a monophyletic clade suggesting its key role in the occurrence of the intestinal infection [15].

Hence, the US study [10] provided the first evidence for an association between the infection by the *Cryptosporidium *firstly named as *Cryptosporidium *CrIT-20 and intestinal infection and pathology in tortoises [14,15].

In summary, a new *Cryptosporidium *genotype was firstly detected and genetically characterized in a marginated tortoise in Italy in 2007 and named *Cryptosporidium *sp. ex *Testudo marginata *CrIT-20 with sequences from two loci deposited in GenBank™ with the Accession numbers EF519704.1 and EF547155.1[14]. No morphological nor biological data on this isolate were generated but the phylogenetic analysis clearly suggested that this *Cryptosporidium *was unique and belonged to the intestinal group [14]. These findings were later on confirmed by the genetic homologies detected in isolates from asymptomatic *C. calyptratus *and *P. regius *from Spain [11], albeit including a single nucleotidic mutation, considered of negligible relevance here, and, more importantly, by the phylogenetic resolution of the *Cryptosporidium *stages isolated from the intestine of *M. tornieri *and *T. horsfieldii *tortoises in USA [15]. On the basis of this knowledge such a *Cryptosporidium *sp. clearly parasitizes different tortoise species, which appear to be the preferential hosts in which it can cause relevant intestinal pathologies. This *Cryptosporidium *has been detected in lizards and snakes also [11], although its pathogenic role in these other reptiles is unknown and it can be argued that the infection is occasional. Although further studies are necessary and more information on the morphology and biology of this new *Cryptosporidium *is required, the multi-locus differences found between *Cryptosporidium *CrIT-20 and of other established *Cryptosporidium *spp., which is a basic taxonomic requirement [3], confirm the statement that this genotype is a distinct species.

Thus, the present biological and molecular data on the *Cryptosporidium *CrIT-20 support to propose it as a new species of *Cryptosporidium *causing intestinal pathologies in tortoises.

**Figure 1:**
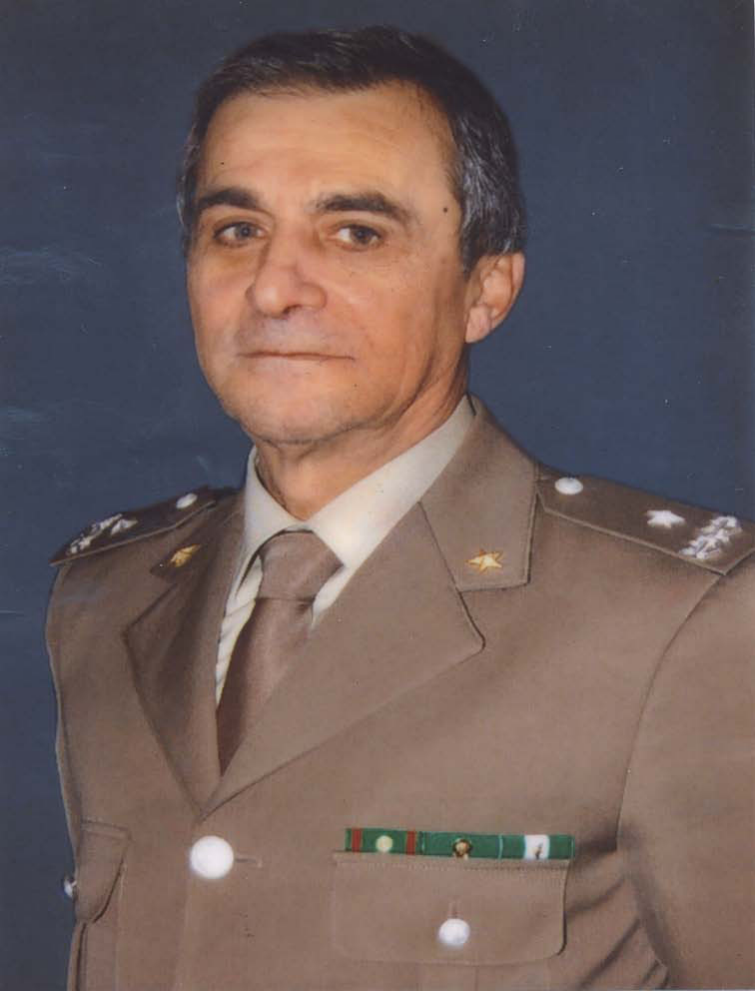
**General Marco Traversa**. Born Marco Traversa on 26^th ^February 1946, in Bari, Italy. On 19^th ^October 1974 he and Maria Calabrese were married and on 15^th ^October 1975 they welcomed their son Donato. Marco began his career in the Italian National Army on 1968 and retired as a General in 2006. Marco died on 4^th ^January 2009 in Bari, Italy.

It is proposed that this *Cryptosporidium *is designated *Cryptosporidium ducismarci *in memory of General Marco Traversa (born Bari, Italy 26^th ^February 1946 - died Bari, Italy 4^th ^January 2009) who served for almost 40 years in the Italian National Army (Figure 1).

In accordance with section 8.6 of the ICZN's International Code of Zoological Nomenclature, copies of this article are deposited at the following five publicly accessible libraries: Natural History Museum, London, UK; American Museum of Natural History, New York, USA; Museum National d'Histoire Naturelle, Paris, France; Russian Academy of Sciences, Moscow, Russia; Academia Sinica, Taipei, Taiwan.

## Abbreviations

SSU: small subunit of the nuclear ribosomal RNA gene; rRNA: nuclear ribosomal RNA; COWP: *Cryptosporidium *oocyst wall protein.

## Competing interests

The author declares that he has no competing interests.
